# Report of the Committee on Medical Education, Appointed by the American Medical Association

**Published:** 1852-06

**Authors:** 


Arn. VI.—*Report of the Committee on Medical Education, appointed by the American Medical Association.* By W. Hooker, M. D.

*The Organizing of the American Medical Association* Read before the Philadelphia County Medical Society, February, 1852. By the President, Samuel Jackson, M. D.

It can not have failed to strike the most casual observer, that a great change has passed upon the character of the medical profession in America. The repose, the stillness, the complacency that marked it at the beginning of the present century are gone, and all is now activity, agitation, restlessness, innovation, and change. Everywhere the cry is for "reform." No one seems satisfied. The feeling is universal, that evils attach to the profession which ought to be remedied; that there are deficiencies in the present mode of medical education which ought to be corrected; that the public mind, so sadly under the dominion of quackery, ought to be enlightened and purified, that medicine may attain to its just rank among the learned professions. Not a few insist that the profession is retrograding. They proclaim that it is not now the lofty calling that it was in former times. They condemn the medical schools, depreciate the preliminary education of medical students, and complain of the estimation in which medical men are held by the community. Doubtless, there is too much complaining. The evils are exaggerated, and representations are made quite unjust to the profession. Medicine is not retrograding. No one who has studied its past history and is acquainted with its present condition, will affirm that it is. It is advancing—amid many difficulties, it is true—hindered by great impediments—marred by great deformities, and bearing along with it numerous evils—but still progressing.

But the discontent on this subject augurs well. It is a necessary condition to improvement; and it can hardly be expected that men, when fired by the zeal of reformers, will not go too far. They will put their case in terms too strong. They are almost sure to exaggerate the errors they attack. But no great harm comes of it, for there are others who go as much too far in the direction of conservatism, and moderate men will take ground between the extreme parties. It would never do to rest where we are. A spirit of self-satisfaction would be fatal. There must be discussion, agitation, a war upon abuses, the demolition of error, strife, revolution, in order to progress. We hail with pleasure therefore the clamor for reform which salutes the ear on every side. It is ominous of good. The tempest will pass, and the sky will be all the more serene for the present din of the elements. The turbid and turbulent flood will run by, and the stream will be found widened, and deepened, and purified by the commotion.

A multitude of plans must be proposed and rejected before the faults complained of will be corrected. Our profession is not to be perfected in a day. We must have patience, and be satisfied to hasten slowly. "Too much importance, we think, has been generally attached to those means which are direct, and too little to those which are indirect in their application. Though these latter have been very commonly lost sight of, we deem them to be quite as essential as the former, in a system of efforts for the improvement of our profession." This is the language of Dr. Hooker, in the Report before us, and we entirely agree with him. We have but little faith in the efficacy of any of the direct means proposed, and the most undoubting confidence that great benefits will flow from the indirect action of the American Medical Association and other medical societies. Decided benefits, the result of its action, are already manifest, as all are ready to acknowledge except those who are too impatient to try any but radical measures.

Dr. Hooker animadverts upon that defect in the education of our medical students which has been so often the subject of complaint—the want of scholastic training. He says—

The laxness of practice which prevails in regard to the preliminary training of medical students, is one of the greatest obstacles in the way of raising the standard of education and attainment in the profession. One practice, which we believe is followed in nearly all our medical schools, has a peculiarly bad influence. We refer to the fact that all students are placed upon the same level in regard to the length of their required term of study before they can be candidates for a degree. There certainly should be a distinction made in this respect between those who have had a deficient preliminary education, and those who have graduated at some literary institution, or who have in some other way duly prepared themselves for the study of medicine. Such a distinction is absolutely necessary to a proper recognition of the importance of a suitable preliminary education, and we therefore recommend that it be adopted at once by all our medical schools.

There are many objections to the innovation here proposed, and we are fully convinced that it will never be carried out by the schools. The distinction would be odious and would not be tolerated by students or their preceptors. Let us see how the principle would operate. About one in twenty, possibly one in ten of the students in our country is a graduate of a literary institution ; the tenth or twentieth would be permitted to take his degree in medicine after two or three years' study, while nine or nineteen would have to wait a year longer. Among that number it is not unreasonable to suppose there would be some his seniors in years, and perhaps much his superiors in natural gifts of mind and habits of study—those who, in the dissecting rooms and the public examinations, would show themselves to be far in advance of him in professional attainments. The young Hunter of mature years and laborious habits, would be obliged to wait, while the favored bachelor of arts took his medical degree on reaching his majority. How long does any one suppose such a rule could be maintained in any medical institution in the land? No; let us insist upon higher literary attainments in our students; impress it upon the public mind continually and in every way, that more preliminary training is necessary; but let us not adopt anything which we should be obliged to abandon in a year. Let us not attempt the impracticable "direct means," but trust to those which, though "indirect in their application," will be far more efficacious in the end.

Dr. Hooker evidently has no very high opinion of lectures as a method of imparting medical instruction; he thinks they induce a listless, passive habit of mind. These are his words on this subject:

The mode of study during eight months of the year ordinarily invites to a listless and careless state of mind, and in the four remaining months the students mind is subjected to such a crowd and pressure of instruction that its powers are wearied out and almost paralyzed. The memory is taxed to the utmost, and there is little time for the exercise of the reflecting and reasoning powers. Such a mass of ill-digested knowledge, of course, in a short time produces satiety, and unfits the mind for anything like active exertion. Even if the student has, during the previous months spent in his preceptor's office, pursued his studies vigorously and systematically, his habits are entirely broken up by this passive and confused reception of such amounts of instruction; and, as he has little time to engage in that critical examination of subjects to which he has accustomed himself, he soon loses the habit of doing so, and loses with it some of that mental vigor which can be preserved and increased only by active exercise.

Now, we had supposed that, as to several branches of medicine, memory was the principal faculty in demand; and as to the others, students, we apprehend, are likely to have quite a sufficient diversity of views presented in most schools, to put in requisition all their "reflecting and reasoning powers." It is not easy to understand how they can become the passive recipients of doctrines as wide apart as the poles. Our own conviction is, that the minds of students are nowhere more vigorously or profitably exercised than in the lecture-room. But upon the principle assumed, the committee are opposed to the extension of the lecture-term. They would favor the extension if, instead of giving lectures, the professors would occupy the added time in examining their students. We incline to the belief, though we have advocated the extension, that the lecture term will continue in most schools, for a good while at least, of its present length. Still, we are in favor of a *five* months' term.

The committee seem to think that much might be done by examinations to excite in a medical class a spirit of emulation, and since these are so much neglected, they do not deem it strange "that listlessness and inattention mark so strongly the majority of the audiences in the lecture-room."

We suspect the committee have been unfortunate in their observations. It is not our experience that such "listlessness and inattention" are characteristic of lecture-rooms. On the contrary, medical students have appeared to us to be the best listeners in the world. We have never seen audiences, under regular didactic discourses, give better attention than has been habitual in the lecture-rooms where we have happened to be. The fault is not that students lack incentives, or that they are careless listeners, but that the period through which they keep up this close application is too short. They have inducements enough to study, and study hard enough till they obtain their diplomas; but unfortunately at that point, with too many of them, study ceases.

There may be a good deal in the following suggestions,

We believe there is; but then they are clearly impracticable. Nothing in the premises can be done directly, but much may result from discussion. The committee are remarking upon the prevalent mode of examination for the medical degree, and go on to say—

Still another difficulty presents itself in attempting a reformation of existing abuses. The schools are, most of them, under the control of corporations which are not in the least degree responsible to the medical profession. They are all independent bodies, and they pursue of course to a great extent, their separate and individual interests. No uniform plan or measure can be adopted, therefore, except by common consent. This we deem to be a radical error in the organization of the medical schools in this country. They should be under the watch and care of the profession. *They should be its property.* They should be among its appliances for securing to the community a body of well educated physicians, and should therefore be, to some extent at least, under its control. The teachers in these schools should be placed there by the profession, and not by corporations that are incapable of judging of their qualifications. Such places of honor and trust should be in the gift of the profession, and should not be at the beck of popular favor. And no school should be established except when the necessity for its existence has been decided upon by the profession.

Exactly in what way a change shall be made which will effect all this we will not prescribe. We propose the whole subject to the consideration of the association, and of the profession; and would only suggest that perhaps the best way to secure so desirable an object would be to have each school responsible to the medical society of the state in which it is located. The State Society also might decide as to the necessity for any new school whenever it should be proposed. And all vacant professorships should be filled by the profession, and one of the means of choice should be something similar to the French Concours. An end should be put to the getting up of schools to gratify private ambition, and to the bestowment of professorships by favoritism, or upon principles of bargain and sale for a certain amount of pecuniary risk in the enterprise. The temple of our science ought not thus to be made a house of merchandise.

The committee continue :

As the result of the lax mode of procedure which marks the system of medical education in this country, the ranks of the profession are everywhere crowded. Statistics show this to be so. For example, in the city of New York, as stated by Dr. Stewart, in the report of 1849, there is one physician to every 500 persons. Deducting the one fourth of these, who are attended gratuitously in one way and another, it leaves but 375 persons to each physician, and a portion of these employ empirics, or take quack medicines.

But the profession is not only crowded, but a large proportion of it is made up of unworthy and ignorant men. So easy is it to obtain a diploma that mere adventurers in great numbers enter the halls of medical science, choosing medicine as a trade, and not as an honorable profession. Such are eager to finish their course of study in the shortest possible time, and are ready, when they have done so, to cope with the most diligent and accomplished of their compeers and the strife for the popular favor and patronage, having received the same honorable badge with them, and perhaps at the same institution. And even quacks, while engaged in the practice of quackery, have been known to receive diplomas from institutions whose representatives appear as delegates in this body.

The only way in which this enormous and motley influx into our profession can be prevented in future is by making a rigid course of training absolutely necessary to the attainment of a degree in all the schools in this country. And in this connection we remark that the objection which is made to a proposed reduction of lecture fees, that it will promote this influx, is in our view groundless. No pecuniary restrictions will be adequate to prevent the evil; but our reliance must be placed upon the strictness of the training to which the student should be subjected, and to the thoroughness of the tests which should be applied to decide upon his fitness for the responsible duties of the physician.

In the last words of this quotation we have the remedy expressed, and the only remedy for the evil within our reach—it is, at last, "*the thoroughness of the tests*, applied" to the applicants for the doctorate. The schools and boards of examination have the remedy in their hands, and they must apply it. It is not in the power of the profession to prevent the multiplication of medical schools, whether established "to gratify private ambition," or for any other purpose; but the public sentiment of the profession tends continually to restrain the schools when established, and must gradually raise the standard of qualification in their graduates. Like individuals, they are amenable to public opinion, and no institution, it may be safely said, can long stand ground against the deliberate judgment of the profession. On all sides it is admitted, that the tests now applied are not rigid enough. The schools must come up to the requisitions of the profession, and we have not a doubt that they are approaching to the standard.

There is matter for reflection in the facts contained in the following extract. It is an undeniable truth, that the mass of the educated talent of our country takes other directions than medicine, especially in the New England States. It is not so much the case in Virginia and the Southern States generally. But everywhere the impression has been too prevalent, that while lawyers and preachers must be men of finished education, if not of bright parts, a doctor could be made out of almost any sort of materials. And it has turned out that though, among medical men, there have been in every generation those the most renowned for learning, there has also been a sad want of scholarship in the profession. Learning, in reality, is not so necessary in medicine as it is in law or theology. A man can not be an eminent lawyer or divine without learning. He can be a very eminent surgeon or physician without it. In the former professions it is indispensable; in medicine it is not. And since it is possible to dispense with it and be respectable, it is too generally dispensed with. Dr. Hooker has been at the pains to collect some statistics on this subject, which are here given:

There is one fact, to which we will call your attention in this connection, that merits the most serious consideration. We refer to the fact that very little of the educated talent of this country finds its way into the medical profession. This we have verified by statistics. But a small proportion of the graduates of our literary institutions, we find, enter the medical profession, in comparison with those who enter the professions of law and divinity. The statistics upon which this statement is based we have gathered from eight colleges in different parts of our country, viz: Harvard, Yale, Dartmouth, Brown, Princeton, Union, Amherst, and Hamilton colleges. In no case have we gone farther back than the year 1800, because there were very few medical schools in existence before that period. We have taken the triennial catalogues of these eight colleges, and, beginning with the first year of the present century, have extended our calculations over a space of about forty-five years. Our statistics include 12,400 graduates. Of these 934, that is, only one in thirteen and a quarter, have become physicians; while 3,211, that is, one in three and four-fifths, have entered the clerical profession. The exact number of those who have chosen the profession of law we are not able to ascertain, because they are not distinguished by any mark from the other graduates; but we are satisfied, from the facts which we have been able to gather on this point, that the proportion is quite as great as that of those who have entered the clerical profession.*

---

*The differences in the results of the statistics of the several colleges, whose catalogues we have examined, are interesting, as showing the influence of various circumstances upon the choice of a profession. We subjoin these results without comment. In Harvard College 1 in 7 1-3 chose medicine, 1 in 6 5-6 chose divinity; in Yale, 1 in 14 7-10 medicine, 1 in 3 7-10 divinity; in Brown, 1 in 9½ medicine, 1 in 2¼ divinity; in Dartmouth, 1 in 18½ medicine, 1 in 3 7-10 divinity; in Princeton, 1 in 16 medicine, 1 in 6⅓ divinity; in Union, 1 in 34¼ medicine, 1 in 3 4-5 divinity; in Hamilton, 1 in 16 3-5 medicine, 1 in 2 3-5 divinity; in Amhurst, 1 in 18 medicine, 1 in 2 divinity.

The reason of the fact which these statistics establish is that *talent is not so sure of its reward in the medical as it is in the other professions.* This explanation, we allow, can not apply to the clerical profession to its full extent, because higher motives than those of ambition are made very prominent in inducing men to enter that profession. But it certainly does apply most fully to the profession of law in comparison with that of medicine.

But there is another fact which at first view seems to be at variance with our explanation of the great fact developed by these statistics. We refer to the fact, of which there is abundance of proof, that the medical profession receives its full share of the *uneducated* talent of the country. If we examine, however, the bearing of this fact, we shall see that, instead of its being inconsistent with our view of the subject, it confirms its truth. While educated talent has been tested, and has ascertained its relative standing, perhaps in the palpable shape of honors, it is far otherwise with uneducated talent. Knowing not its powers, and having never received any rewards of its efforts, it is not so calculating and far-sighted on these points, and more readily follows where inclination and taste lead it. And as the door of the medical profession is widely opened, and as uneducated talent sees that it can cope with educated talent with more hope of success than it can in other fields of effort, it is attracted more largely to this than it is to the other professions.

But we have other statistics which reveal a fact confirmatory of the view which we have given of this subject. We have found that of those who have received honors in our collegec, the proportion that has entered the medical profession is smaller than that which has entered it from the whole body of graduates. Thus in Harvard, while one in seven and one-third of the graduates has be-

---

We are aware that these statistics are not strictly correct. In making up the Triennial Catalogues, the profession which is chosen by the graduates is not in every instance ascertained. Still the omissions from this cause, we are satisfied, are not sufficient to affect materially the general result at which we have arrived. The defect is probably greater in the case of Union College than in any other, in relation to those who are designated as having chosen the medical profession.

come a physician, but one in eleven and a half of the members of the Phi Beta Kappa Society (which generally comprises the best third of each class in point of talent and acquirement) has chosen our profession. In Yale College, also, our profession was chosen by one in about fourteen and a half of all the graduates, but by only one in eighteen and a quarter of the members of the Phi Beta Kappa Society. And of 333 who took the highest honors in twenty-three classes, but one in thirty and a half became a physician—showing that the more we sift the classes in our colleges in regard to talent, the less is the proportion of these who enter the medical profession. A similar result is found in the statistics of Hamilton College. While the medical profession was chosen by one in sixteen and three-fifths of the graduates as a body, it was chosen by only one in twenty-five of those who won the honors in the several classes of that institution.

The import of the state of things developed by the above statistics is augmented by the fact that the medical profession embraces a much larger body of men than either of the other professions. Even if the number of educated men who become physicians was equal to the number of those who choose the profession of law or divinity, the proportion of educated to uneducated talent would be much less in the medical than in the other professions.

We think it must be admitted by even the most sanguine friends of reform, that the evils deplored in this extract from the report of the committee are such as are not to be speedily remedied by the action of medical associations. They are to some extent inherent in our profession—necessary evils—and not to be eradicated by any species of legislation. As long as law or any other vocation holds out greater profits or honors to the youthful aspirants, so long will those callings be preferred to medicine. But so far as these grievances are susceptible of remedy, they are not to be reached by those of a direct or radicial character; they can be corrected by nothing short of an enlightened public sentiment.

Writers on this subject, we have thought, are very prone to fall into the style of those enthusiasts often met with, who are persuaded that they are in possession of some specific remedy—some panacea—before which the ills that flesh is heir to yield at once, as the dark things of night fly away at the coming of the sun. "Only let the association give their fiat," say they, "and the work will soon be done." It is as simple and facile as the process of *Puck* in the *Midsummer Night's Dream*—"the man shall have his mare again and all shall be well." We have said that we rest many hopes upon the association, and look to it for important results, but we confess our faith does not extend so far as this. Its influence is eminently benign, and will continue to be felt more widely and beneficially, but its "fiats," we suspect, will not amount to much. It is a congress, indeed, but without any power to make laws. It may legislate much, but its legislation is not binding, further than as it embodies public opinion. The public opinion of the profession is omnipotent.

But a profound writer remarked a good while ago, that "one of the hardest things in the world to get at, is the truth." It is not an easy matter to gather the public opinion. The Association is already complained of for not reflecting it fairly, and the object of Dr. Jackson's pamphlet, the title of which we have placed at the head of this article, is to bring about its reorganization. Dr. Jackson proposes that the officers of all public institutions, as such, shall be excluded from the association, and the delegation be made up entirely of the members of county societies; and this, we suppose, was discussed, along with many kindred projects, at the last meeting. We do not propose to discuss it here, but we can not help remarking, that it is another symptom of that restlessness which so extensively pervades the profession; that striving after something better; that desire of innovation and change. This spirit must be watched, or it will add another to the multitudinous evils of which we now complain. The association is in constant danger of acquiring a political cast—of becoming a great primary assembly for the discussion of medical politics. In the estimation of some members the last meeting was rendered less profitable and interesting by the prevalence of this spirit, in which the higher interests of science seemed for the time to be somewhat overlooked. Medical politics must continue to be a leading object with the association, but they ought to be restrained within reasonable bounds. They ought not to be allowed to swallow up every thing else. Let Dr. Jackson's amendment be canvassed; let all the other medical politicians bring their amendments in; let us have the widest possible range of discussion, the utmost freedom of opinion and speech. *E collisione scintilla.* Light will come of the collision. Many favorite projects, many wise schemes, must fare badly, but the profession will be advanced and benefited.

